# A novel lipid metabolism-based risk model associated with immunosuppressive mechanisms in diffuse large B-cell lymphoma

**DOI:** 10.1186/s12944-024-02017-z

**Published:** 2024-01-22

**Authors:** Zhaoli Zhang, Chong Zhao, Shaoxin Yang, Wei Lu, Jun Shi

**Affiliations:** Department of Hematology, Shanghai Ninth People’s Hospital, Shanghai Jiaotong University School of Medicine, Shanghai, China

**Keywords:** Diffuse large b-cell lymphoma, Lipid metabolism, Prognosis, Immune infiltration

## Abstract

**Background:**

The molecular diversity exhibited by diffuse large B-cell lymphoma (DLBCL) is a significant obstacle facing current precision therapies. However, scoring using the International Prognostic Index (IPI) is inadequate when fully predicting the development of DLBCL. Reprogramming lipid metabolism is crucial for DLBCL carcinogenesis and expansion, while a predictive approach derived from lipid metabolism-associated genes (LMAGs) has not yet been recognized for DLBCL.

**Methods:**

Gene expression profiles of DLBCL were generated using the Gene Expression Omnibus (GEO) and The Cancer Genome Atlas (TCGA) databases. The LASSO Cox regression was used to construct an effective predictive risk-scoring model for DLBCL patients. The Kaplan-Meier survival assessment was employed to compare a given risk score with the IPI score and its impact on the survival of DLBCL patients. Functional enrichment examination was performed utilizing the KEGG pathway. After identifying hub genes via single-sample GSEA (ssGSEA), immunohistochemical staining and immunofluorescence were performed on lymph node samples from control and DLBCL patients to confirm these identified genes.

**Results:**

Sixteen lipid metabolism- and survival-associated genes were identified to construct a prognostic risk-scoring approach. This model demonstrated robust performance over various datasets and emerged as an autonomous risk factor for predicting the development of DLBCL patients. The risk score could significantly distinguish the development of DLBCL patients from the low-risk and elevated-risk IPI classes. Results from the inhibitory immune-related pathways and lower immune scores suggested an immunosuppressive phenotype within the elevated-risk group. Three hub genes, *MECR*, *ARSK*, and *RAN*, were identified to be negatively correlated with activated CD8 T cells and natural killer T cells in the elevated-risk score class. Ultimately, it was determined that these three genes were expressed by lymphoma cells but not by T cells in clinical samples from DLBCL patients.

**Conclusion:**

The risk level model derived from 16 lipid metabolism-associated genes represents a prognostic biomarker for DLBCL that is novel, robust, and may have an immunosuppressive role. It can compensate for the limitations of the IPI score in predicting overall survival and has potential clinical application value.

**Supplementary Information:**

The online version contains supplementary material available at 10.1186/s12944-024-02017-z.

## Introduction

Diffuse large B-cell lymphoma (DLBCL) has the highest prevalence of any class of non-Hodgkin lymphoma [1]. In spite of the high cure rates associated with DLBCL, outcomes exhibit significant variation, often due to the heterogeneity observed at the clinical, pathological, and molecular levels [2–4]. Accurate prediction and risk stratification are imperative for selecting appropriate treatment approaches. The International Prognostic Index (IPI) scoring approach, is comprised of five clinical factors: age, Eastern Cooperative Oncology Group (ECOG) status, Ann Arbor stage, lactate dehydrogenase (LDH) quantity, and extranodal site involvement, has been used for over two decades and is endorsed by multiple reports [5]. However, the aggregate incidence of disease progression after five years for individuals with a low-risk IPI varies from 15 to 22%, while the 5-year overall survival (OS) level for patients possessing an elevated-risk IPI ranges from 49 to 59%. These statistics underscore that some patients do not align with the expected early or late-stage disease categories [6, 7]. Integrating molecular and other tumor immune microenvironment features into current clinical scoring approaches is a promising avenue.

Dysregulated lipid breakdown and usage are critical for the growth and expansion of tumors, with increasing evidence highlighting its essential reprogramming in tumors [8–11]. This reprogramming is progressively recognized as a novel key characteristic of tumor malignancy. Clinical investigations have demonstrated the use of statins as a practical approach for the reduction of occurrence risk and an increase in the response rate to DLBCL chemotherapy [12, 13], suggesting the involvement of lipid metabolism in DLBCL onset and chemoresistance. Furthermore, altered fatty acid metabolism has been suggested as a significant oncogenic factor in DLBCL [14]. Overexpression of fatty acid synthase (FASN) and fatty acid translocatase CD36 have been linked to chemotherapy sensitivity and limited clinical projection for DLBCL [15–17]. While numerous studies have documented the predictive potential of risk characteristics derived from lipid metabolism-associated genes (LMAGs) across various solid tumors [18–22], the utility of LMAG-based risk models in characterizing DLBCL subtypes as well as the development of disease has remained undefined.

Lipids not only support cancer progression via energy production and lipid accumulation in tumor cells but also exert influence on the tumor immune microenvironment through interactions with stromal and immune cells [10]. In this study, available datasets were utilized for the construction and validation of DLBCL prognostic characteristics according to LMAGs. Furthermore, an LMAG-derived risk model was developed to improve the risk stratification provided by the IPI score in DLBCL. Pathway analysis and immune infiltration assessment revealed that *MECR*, *RAN*, and *ARSK* were associated with an immunosuppressive tumor immune microenvironment in the elevated-risk score class. This investigation aimed to identify potential LMAGs with diagnostic value and establish a potential risk model, serving as possible clinically significant biomarkers and providing a reference for the identification and development of DLBCL.

## Methods

### Data source

Six human lipid metabolism pathways were acquired from the Molecular Signature Database (MSigDB) [23]. These pathways encompass peroxisome proliferator-activated receptor alpha, metabolism of lipids, transcriptional regulation of white adipocyte differentiation, sphingolipid metabolism, glycerophospholipid metabolism, and fatty acid metabolism. From these lipid metabolism pathways, 776 genes linked to lipid metabolism were extracted (Supplementary Table 1). A total of four cohorts with clinical characteristics related to DLBCL patients from the Gene Expression Omnibus (GEO) database and The Cancer Genome Atlas (TCGA) database were obtained [24, 25]. Specifically, the GSE181063 cohort was designated as the training cohort since it contains the largest sample size, while the remaining datasets, GSE10846, GSE11318, and TCGA-NCICCR, served as validation cohorts. Patients lacking complete expression and clinical data were excluded, resulting in an analysis of 644 patients from GSE181063, 233 patients treated with R-CHOP (rituximab combined with cyclophosphamide, doxorubicin, vincristine, and prednisolone) from GSE10846, 181 patients treated with CHOP from GSE10846, 234 from TCGA-NCICCR, and 163 from GSE11318 (Table 1). A total of 523 genes associated with lipid metabolism from the expression profiles of these four cohorts as candidate genes were utilized in this study (Supplementary Table 1).

**Table 1:** Clinical pathological characteristics of DLBCL cases identified in the training and validating datasets

Clinical features		Training cohort	Validating cohorts			
		GSE181063 (*n* = 644)	NCICCR (*n* = 234)	GSE10846 RCHOP(*n* = 233)	GSE10846 CHOP (*n* = 181)	GSE11318 (*n* = 163)
**Tissue**		unknown	lymph node	lymph node	lymph node	lymph node
**Firstline regimen**		RCHOP	RCHOP or CHOP	RCHOP	CHOP	CHOP
**Event**						
0		354(54.97%)	136(58.12%)	173(74.25%)	76(41.99%)	67(41.10%)
1		290(45.03%)	98(41.88%)	60(25.75%)	105(58.01%)	96(58.90%)
**Gender**						
Female		326(50.62%)	95(40.60%)	99(42.49%)	73(40.33%)	73(44.79%)
Male		318(49.38%)	139(59.40%)	134(57.51%)	90(49.72%)	90(55.21%)
unknown		0	0	0	18(9.94%)	0
**Age**						
≤ 60		190(29.50%)	116(49.57%)	113(48.50%)	75(41.44%)	69(42.33%)
> 60		454(70.50%)	118(50.43%)	120(51.50%)	106(58.56%)	94(57.67%)
**ECOG, PS**						
< 2		473(73.45%)	163(69.66%)	158(67.81%)	138(76.24%)	122(74.85%)
≥ 2		121(18.79%)	49(20.94%)	52(22.32%)	41(22.65%)	39(23.93%)
unknown		50(7.76%)	22(9.40%)	23(9.87%)	2(1.10%)	2(1.23%)
**Ann Arbor stage**						
I + II		223(34.63%)	110(47.01%)	105(45.06%)	83(45.86%)	75(46.01%)
III + IV		351(54.50%)	121(51.71%)	121(51.93%)	97(53.59%)	87(53.37%)
unknown		70(10.87%)	3(1.28%)	7(3.00%)	1(0.55%)	1(0.61%)
**LDH**						
>UNL		327(50.78%)	105(44.87%)	93(39.91%)	85(46.96%)	76(46.63%)
≤UNL		193(29.97%)	97(41.45%)	99(42.49%)	74(40.88%)	68(41.72%)
unknown		124(19.25%)	32(13.68%)	41(17.60%)	22(12.15%)	19(11.66%)
**Cell of origin**						
GCB type		306(47.52%)	110(47.01%)	107(45.92%)	76(41.99%)	66(40.49%)
ABC type		179(27.80%)	82(35.04%)	93(39.91%)	74(40.88%)	70(42.94%)
MHG type		45(6.99%)	0	0	0	0
UNC type		114(17.70%)	42(17.95%)	33(14.16%)	31(17.13%)	27(16.56%)
**B symptoms**						
Yes		247(38.35%)	unknown	unknown	unknown	unknown
No		397(61.65%)	unknown	unknown	unknown	unknown
**IPI score**						
0–2		277(43.01%)	126(53.85%)	unknown	unknown	unknown
3–5		208(32.30%)	67(28.63%)	unknown	unknown	unknown
unknown		159(24.69%)	41(17.52%)	unknown	unknown	unknown
**Extranodal involvement**						
Yes		254(39.44%)	111(47.44%)	116(49.79%)	29(16.02%)	28(17.18%)
No		305(47.36%)	109(46.58%)	87(37.34%)	151(83.43%)	134(82.21%)
unknown		85(13.20%)	14(5.98%)	30(12.88%)	1(0.55%)	1(0.61%)

Abbreviations: ECOG, Eastern Cooperative Oncology Group; PS, performance status; GCB, germinal center B-cell like; ABC, activated B-cell like; MHG, molecular high-grade; UNC, unclassified; IPI, International Prognostic Index; LDH, lactate dehydrogenase; UNL, upper limit of normal

### Construction and confirmation of the forecast model

Univariate Cox regression examination was conducted to find genes with prognostic value from the 523 candidate genes. To circumvent over-fitting, the “glmnet” package was employed to analyze the prognosis-related LMAGs via least absolute shrinkage and selection operator (LASSO) Cox regression analysis. Hazard ratios (HR) > 1.2 and HR < 0.8 were identified as cutoff points. Subsequently, a lipid-associated prognostic risk-scoring approach was established via multivariate Cox regression. To ensure its robustness, internal confirmation of the final multivariate model was conducted by employing bootstrapping with 1000 bootstrap samples. This process yielded shrinkage factors for fine-tuning regression coefficients and adjusted model intercepts. These adjustments were then applied to prediction formulas and helped evaluate model performance, considering optimism-corrected measures. For each patient, the risk score was computed using the following formula:

$$RiskScore = \sum\nolimits_{{\text{i}} = 1}^n {Coef\left( {mRN{A_i}} \right)} \times Expression\left( {mRN{A_i}} \right)$$

According to the median risk score, DLBCL patients were separated into low- and elevated-risk classes. Subsequently, the variability in overall persistence across these two classes was examined using Kaplan-Meier curves as well as a log-rank test. Ultimately, the time-dependent receiver operating characteristic (ROC) curve examination was employed to determine the specificity and sensitivity of the risk signature utilizing the “survivalROC” R package. The area under the curve (AUC) value was determined and used to designate the effect of ROC.

### Development and assessment of a nomogram according to the risk level approach

Cox regressions using both univariate and multivariate data were employed to obtain independent prognostic factors according to the risk-scoring model as well as the IPI score from the GSE181063 dataset. Subsequently, a nomogram was generated through the use of the “rms” R package using independent prognostic factors. The predictive effect of this nomogram was examined through the concordance index (C-index) as well as calibration plots. A C-index of 0.5 demonstrates the lack of predictive discrimination, while a C-index value of 1.0 suggests an ideal separation of patients possessing different prognoses. Calibration plots were employed to assess the nomogram prediction probabilities in comparison to the observed rates. The nomogram was subsequently confirmed using the TCGA-NCICCR dataset.

### Functional enrichment analysis

To investigate the differences in possible KEGG pathways enriched between high- and low-risk patients, gene set variation analysis (GSVA) was conducted utilizing the clusterProfiler package on four datasets. “c2.cp.kegg.v7.5.1.entrez. gmt” was obtained as the reference gene set. Furthermore, the differentially expressed genes (DEGs) over the two groups were examined, and gene set enrichment analysis (GSEA) was conducted. The condition was perceived as enriched in situations in which the nominal (NOM) *P-*value < 0.05, false discovery rate (FDR), q value < 0.25, and the normalized enrichment score (|NES|) > 1. Single sample GSEA (ssGSEA) analysis was conducted on specified KEGG pathways using the GSVA R package, and correlation analysis was conducted between KEGG pathways and LMAGs expression information.

### Immune infiltrating analysis

The patient’s ESTIMATE Score, Immune Score, and Stromal Score were acquired using an estimate package to predict the infiltration of stromal and immune cells into the tumor immune microenvironment. Analysis using ssGSEA was conducted on specific immune cells utilizing the GSVA package to examine the immunological characteristics of the elevated-risk and low-risk score groups.

### Immunohistochemical and immunofluorescence staining

Immunohistochemistry, as well as immunofluorescence on lymph node biopsies from DLBCL patients, were conducted as outlined previously [26]. The sections were stained using a primary antibody (anti-MECR, Proteintech, Cat. NO: 51027-2-AP; anti-RAN, Proteintech, Cat. NO: 67500-1-Ig; anti-ARSK, Bioss, Cat. NO: bs-9102R; anti-CD3, Proteintech, Cat. NO: 60181-1-Ig; anti-CD20, Proteintech, Cat. NO: 60271-1-Ig). The nucleus was stained using DAPI (Solarbio, Beijing, China) for use in immunofluorescence. Images of stained slides for these markers were scanned at 400× magnification using an optical microscope (Olympus Co., Tokyo, Japan). Immunohistochemistry results were quantified by counting the area of positive signals using Image J software. Fluorescent images were captured via a confocal laser microscopy system (Leica SP2).

### Statistical analyses

Statistical data were analyzed using R software (version 4.2.1) and GraphPad Prism 8 software (GraphPad, Inc., USA). Kendall rank correlation was used to estimate relations between IPI score and lipid metabolism**-**based risk levels. Comparisons between groups were conducted through the use of the Student’s *t*-test. Survival curves were plotted based on the Kaplan-Meier method. *P* < 0.05 indicated the significance.

## Results

### Construction of a lipid metabolism-based risk score model for DLBCL patients

**Fig. 1:**
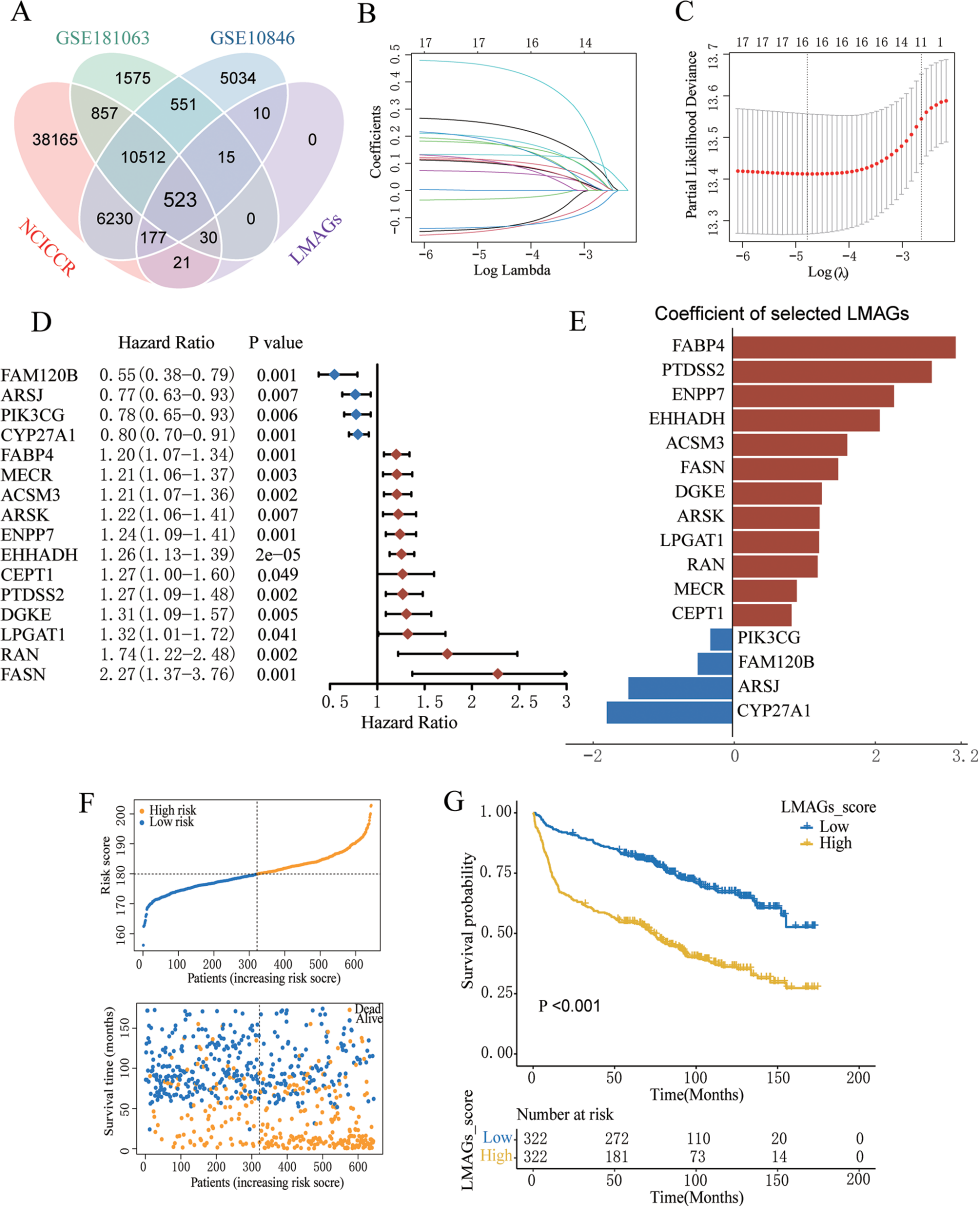
Development of the lipid metabolism-based risk level approach for DLBCL patients. **(A)** Authentication of 523 lipid metabolism-related genes in three datasets (GSE181063, GSE10846, and NCICCR) using Venn diagrams. Changes in color denote differences in datasets. **(B)** LASSO coefficients of 16 obtained LMAGs over the 10-fold cross-validation approach. Vertical dotted lines denote the optimal values utilizing the minimum and 1-SE criteria. **(C)** Partial likelihood variance was uncovered using the LASSO regression model as well as the 10-fold cross-validation. Vertical dotted lines denote the optimal values utilizing the minimum and 1-SE criteria. **(D)** Forest plot of the linkages between the infiltrating levels of 16 prognostic molecules as well as the OS of the training cohort. The HR, 95% CI, and *P*-value were computed using univariate Cox regression analysis. **(E)** Coefficients for the 16 prognostic molecules within the Cox regression model. **(F)** The risk score distribution and survival levels of 16-gene signatures from the GSE181063 dataset. **(G)** Survival curves across the two risk groups from the GSE181063 dataset

To identify prognostic genes for DLBCL, four cohorts with clinical information and overall survival data from the GEO and TCGA databases were screened. By intersecting with lipid metabolism pathways, 523 LMAGs were selected (Fig. 1A). The GSE181063 cohort, containing the largest sample size, was utilized as a training set for the establishment of the predictive approach. Following LASSO Cox regression analysis, 16 LMAGs possessing the most elevated normalized enrichment levels were selected from these 523 LMAGs for the building of the lipid metabolism-associated risk level model in the GSE181063 dataset (Fig. 1B and C). The forest plot demonstrates the relationship between the expression quantities of these 16 LMAGs and overall survival (Fig. 1D). Notably, *ACSM3*, *ARSK*, *CEPT1*, *DGKE*, *EHHADH*, *ENPP7*, *FABP4*, *FASN*, *LPGAT1*, *MECR*, *PTDSS2*, and *RAN* were identified to be significantly associated with a negative outcome, whereas *ARSJ*, *CYP27A1*, *FAM120B*, and *PIK3CG* exhibited the opposite effect. Kaplan-Meier analysis confirmed the prognostic significance of these 16 LMAGs in DLBCL (Supplementary Fig. 1), underscoring their individual roles in DLBCL prognosis.

To construct the risk score model for DLBCL, coefficients for the 16 LMAGs were identified using a Cox regression model (Fig. 1E). Subsequently, the lipid metabolism-related risk score was computed for individual patients within the training set. Individuals with DLBCL were stratified into low- and elevated-risk categories according to the median risk score. Visualization of risk score distribution and survival status in this dataset is outlined in Fig. 1F. Furthermore, the Kaplan-Meier analysis determined that the elevated-risk group was associated with significantly reduced overall survival compared to the reduced-risk group within the training set (Fig. 1G). When compared to single-gene models, the risk score model, according to the 16 LMAGs, possessed superior predictive efficiency, as indicated by the C-index and AUC values (Supplementary Fig. 2A and B).

### Reliable validation of the risk score model across diverse groups

**Fig. 2:**
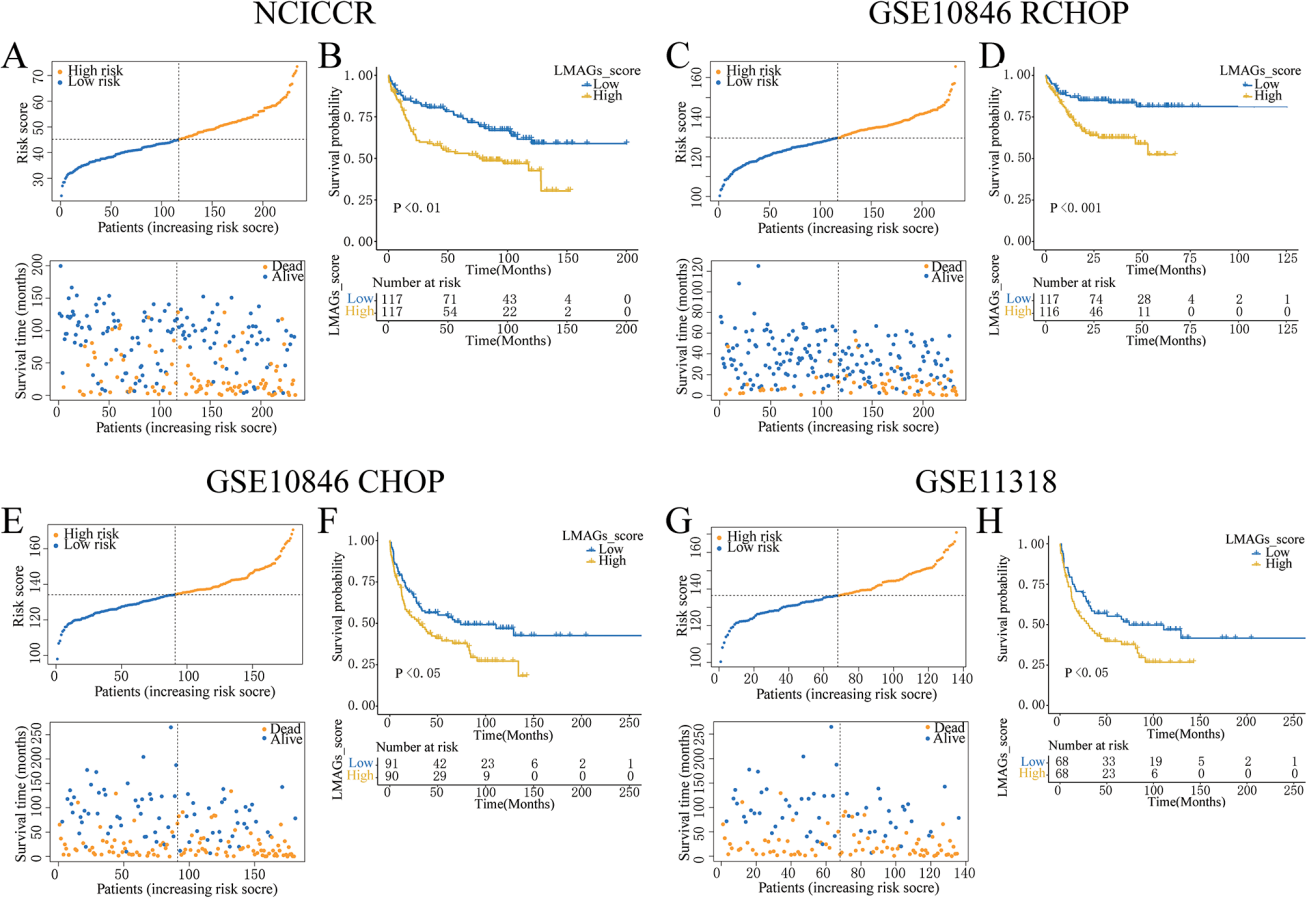
Robust confirmation of risk score approach in testing cohorts. Division of risk score and survival status of 16-gene signatures from the NCICCR dataset **(A)**, GSE10846 R-CHOP dataset **(C)**, GSE10846 CHOP dataset **(E)**, and GSE11318 dataset **(G)**. Survival curves across two risk classes in the NCICCR dataset **(B)**, GSE10846 R-CHOP dataset **(D)**, GSE10846 CHOP dataset **(F)**, and GSE11318 dataset **(H)**

In order to examine the reliability of the model, identical coefficients were applied to internal testing cohorts, such as TCGA-NCICCR, GSE10846, and GSE11318 cohorts. Given the significant improvement in DLBCL prognosis upon rituximab plus polychemotherapy (R-CHOP) in the era of immunotherapy, the GSE10846 cohort was further divided into GSE10846-RCHOP and GSE10846-CHOP according to the treatment received. The division of risk scores as well as survival across these cohorts is indicated in Fig. 2A, C, E, and G. Remarkably, patients possessing high-risk levels exhibited substantially elevated levels of death compared to those possessing low-risk scores, consistent with training set findings. Kaplan-Meier examination verified the significant prognostic differences between elevated-risk and reduced-risk classes across the entirety of the testing cohorts (Fig. 2B, D, F, and H). These results highlight the robustness of the lipid metabolism-based risk score model, which retains stable prognostic predictive capability across various cohorts, including those containing immunotherapy-treated patients.

### The risk score approach can be a unique predictor in DLBCL

Given that various clinical characteristics may obstruct the risk score, patients from the GSE181063 dataset were divided into several classes based on age, cell of origin, ECOG grade, extranodal involvement, gender, IPI, Ann Arbor stage, B symptoms, or LDH. Regardless of the clinical subgroups, the reduced-risk group consistently exhibited increased survivorship probabilities compared to the elevated-risk group (Supplementary Fig. 3, *p* < 0.05), confirming independence in our risk level model relative to clinical characteristics as well as its reliable predictive power for DLBCL survival.

Various IPI scores were compared to the lipid metabolism-based risk score via Cox regression analysis. In the GSE181063 dataset, it was found that the risk score ranged from a minimum of 156 to a maximum of 203, with an average of 180. A similar pattern was observed in the NCICCR dataset. Therefore, the change in mortality risk among the study population was evaluated when the risk score increases by 10 units. Analysis utilizing GSE181063 and TCGA-NCICCR datasets (the only datasets with available IPI score information) indicated that each 10 units increase in the lipid metabolism-based risk score was tied to a corresponding relative mortality risk of 1.934 (95% CI: 1.577–2.373) in the GSE181063 dataset and a relative risk of 1.290 (95% CI: 1.010–1.647) in the TCGA-NCICCR dataset (Tables 2 and 3). To further verify the independence of the lipid metabolism-based risk score model, the association between risk score and the IPI score was analyzed. As shown in Table 4, the level of risk score showed a weak positive correlation with the IPI score in the datasets. These findings verified the lipid metabolism-based risk score model was an independent predictor of overall survival in DLBCL patients.

**Table 2:** Cox analysis of lipid metabolism-based risk scores as well as the IPI for the overall survival of patients with DLBCL from the GSE181063 cohort

	Univariate Cox Regression		Multivariate Cox Regression	
Variables	HR (95% CI)	*P* value	HR (95% CI)	*P* value
**IPI**				
IPI 0–1	0.187 (0.124–0.282)	< 0.001	0.226 (0.149–0.342)	< 0.001
IPI2	0.335 (0.229–0.489)	< 0.001	0.344 (0.235–0.503)	< 0.001
IPI3	0.432 (0.294–0.610)	< 0.001	0.438 (0.303–0.632)	< 0.001
IPI 4–5	1 (reference)		1 (reference)	
**LMAGs_RiskScore, per 10 units**	2.101 (1.722–2.563)	< 0.001	1.934 (1.577–2.373)	< 0.001

**Table 3:** Cox analysis with lipid metabolism-based risk levels as well as the IPI for the overall survival of patients with DLBCL from the NCICCR cohort

	Univariate Cox Regression		Multivariate Cox Regression	
Variables	HR (95% CI)	*P* value	HR (95% CI)	*P* value
**IPI**				
IPI 0–1	0.177 (0.093–0.339)	< 0.001	0.209 (0.108–0.407)	< 0.001
IPI2	0.340 (0.178–0.653)	0.001	0.371 (0.192–0.715)	0.003
IPI3	0.510 (0.272–0.958)	0.036	0.544 (0.288–1.027)	0.060
IPI 4–5	1 (reference)		1 (reference)	
**LMAGs_RiskScore, per 10 units**	1.456 (1.159–1.829)	0.001	1.290 (1.010–1.647)	0.041

**Table 4:** Association of lipid metabolism-based risk levels with IPI score

	LMAGs_RiskScore			
	GSE181063		NCICCR	
	Kendall’s tau-b	*P* value	Kendall’s tau-b	*P* value
**IPI score**	0.192	< 0.001	0.194	< 0.001
0–1				
2				
3				
4–5				

### Comparison of the lipid metabolism-based risk level as well as IPI score

**Fig. 3:**
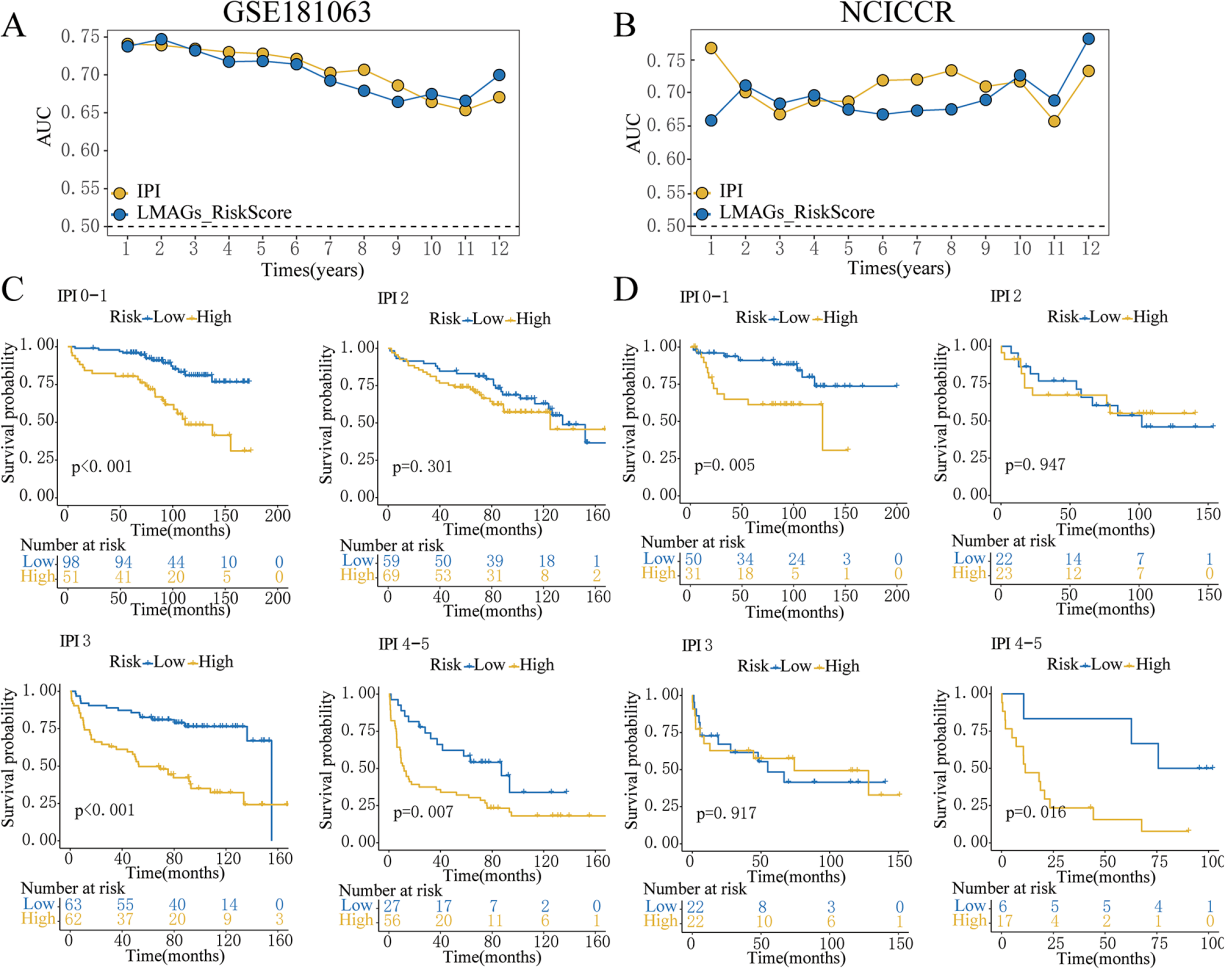
Comparison of the lipid metabolism-based risk level and IPI score. **(A-B)** AUC values of risk score and IPI score over the course of 12 years from the GSE181063 and NCICCR datasets. **(C-D)** Kaplan-Meier curves denoting OS between the high- and low-risk groups from DLBCL patients possessing different IPI scores from the GSE181063 and NCICCR datasets

In order to examine the respective impacts lipid metabolism-based risk scores and the IPI score have on DLBCL prognostic accuracy, a time-dependent ROC analysis utilizing the GSE181063 and TCGA-NCICCR datasets was conducted. As illustrated in Fig. 3A and B, no significant difference was observed concerning the AUC from 2 to 5 years. However, from 5 to 10 years, the AUC for the IPI score surpassed that of the lipid metabolism-derived risk level, especially within the TCGA-NCICCR data. Interestingly, after 10 years, the AUC for the lipid metabolism-based risk score outperformed the IPI score. Kaplan-Meier plots of OS demonstrated that the lipid metabolism-associated risk level was able to significantly distinguish the prognosis of DLBCL patients possessing a reduced-risk IPI (score = 0–1) and an elevated-risk IPI (score = 4–5) in both the GSE181063 and TCGA-NCICCR datasets (Fig. 3C and D). However, no significant prognostic change was observed between the high-risk score class and the low-risk score class among DLBCL patients with IPI score = 2 or IPI score = 3. These findings indicate that a lipid metabolism-derived risk score approach is able to compensate for the limitations of the IPI score in predicting OS, particularly in low-risk and high-risk IPI groups.

### Establishment of a nomogram combined lipid metabolism-based risk score with IPI score

**Fig. 4:**
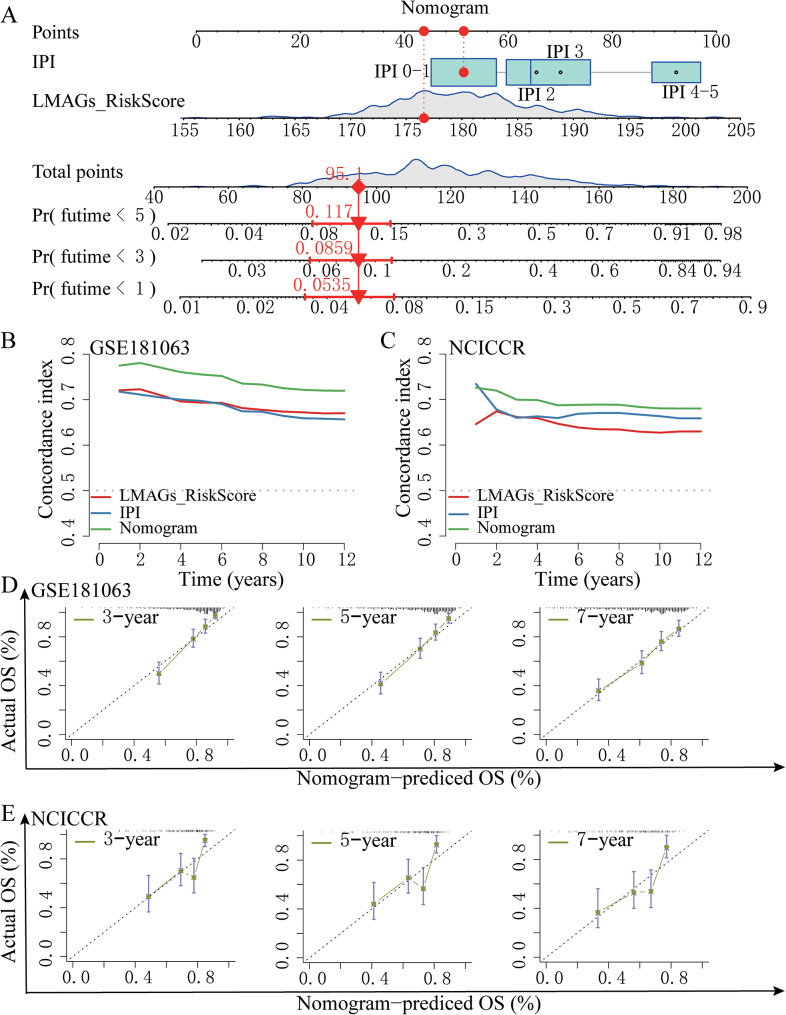
Creation of a nomogram combining the risk score with the IPI score. **(A)** The 1-year, 3-year, and 5-year survivability of DLBCL patients was predicted by a nomogram based on their risk scores, IPI, and total points. **(B-C)** Time-dependent C-index chart for the nomogram as well as various clinical factors from the GSE181063 and NCICCR datasets. (D-E) Calibration plots used for prediction in DLBCL patients with 3-, 5-, and 7-year OS in the GSE181063 and NCICCR datasets. X-axis showed the nomogram-predicted survivability, while y-axis displayed the actual survivability

To enhance the reliability and accuracy of prognostic predictions, a predictive nomogram integrating the risk score model as well as the IPI score was developed (Fig. 4A). Each patient received a total point score through addition of the points given for each element, with a higher number correlating with more negative outcomes. Time-dependent C-index curves in the GSE181063 and TCGA-NCICCR cohorts uncovered that the nomogram exhibited the highest index among all variables, outperforming discrete factors (Fig. 4B and C). Moreover, calibration curves exhibited substantial agreement across the nomogram-derived probabilities of three-, five-, and seven-year OS and the true OS in both the GSE181063 (Fig. 4D) and TCGA-NCICCR datasets (Fig. 4E). These results confirm the reliability and accuracy of the nomogram using lipid metabolism-based signature risk scores for the prediction DLBCL development.

### Functional enrichment examination of the lipid metabolism-based risk level model

**Fig. 5:**
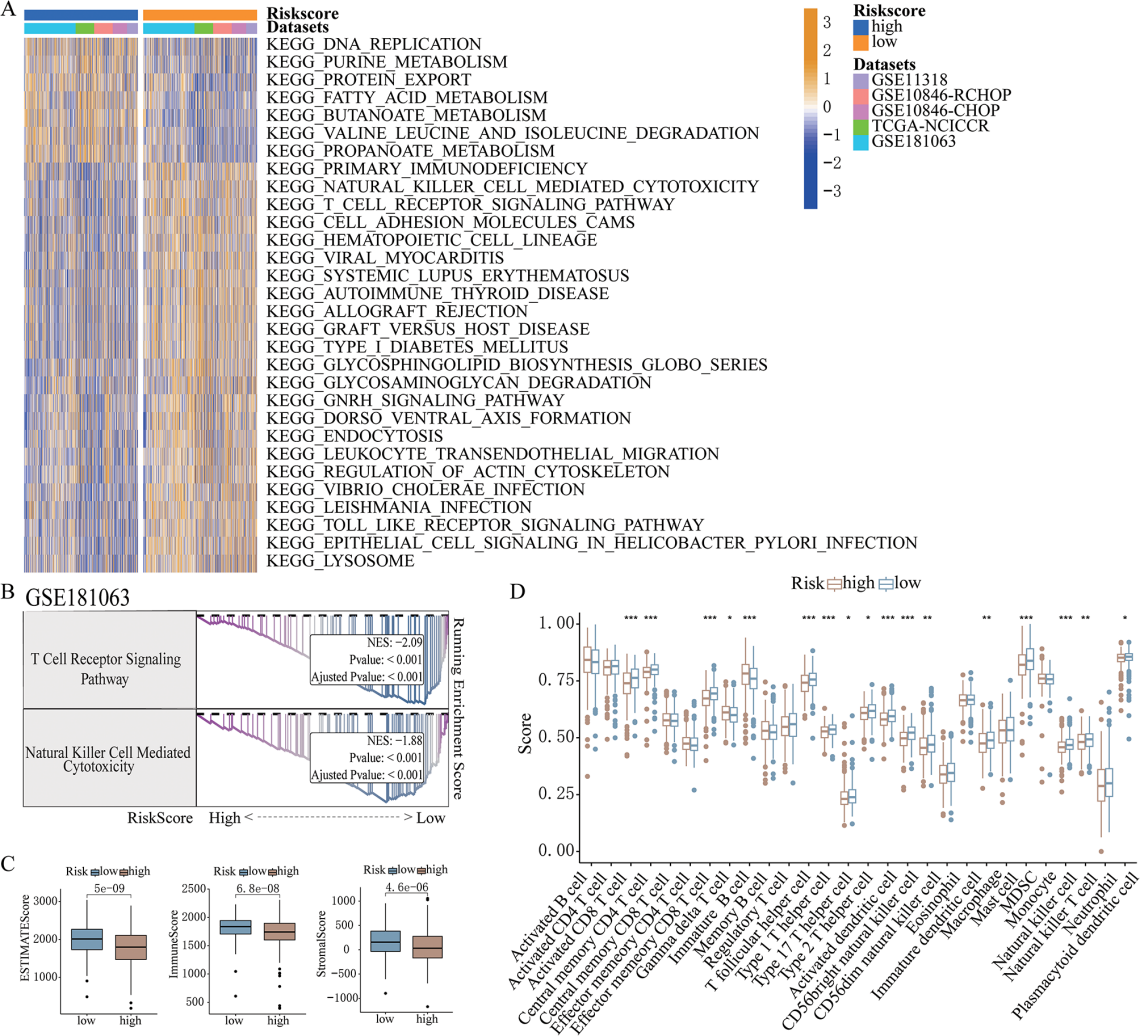
Functional enrichment examination of the lipid metabolism-derived risk level approach. **(A)** GSVA examination of the biological pathways within the high- and low-risk score groups from the GSE181063, GSE10846 R-CHOP, GSE10846 CHOP, GSE11318, and NCICCR datasets. Orange and blue indicates the activation and inhibition of biological pathways, respectively. **(B)** GSEA indicates a significant increase in natural killer cell-mediated cytotoxicity, and T-cell receptor signaling pathways in the GSE181063 cohort. **(C)** Estimate score, immunity score, and stromal score across the high- and low-risk groups from the GSE181063 cohort. **(D)** ssGSEA comparison of the scores from various infiltrating immunity cells across DLBCL patients with high- and low-risk scores from the GSE181063 cohort. ***P < 0.001; **P < 0.01; *P < 0.05

In order to elucidate the underlying methods contributing to the different findings identified by the risk score model, a KEGG pathway examination was performed. The findings of this analysis revealed that the most significantly activated pathways in the high-risk class were related to metabolism, including fatty acid metabolism, glucose metabolism, and amino acid metabolism (Fig. 5A). Conversely, immune-related pathways including natural killer cell-mediated cytotoxicity, T-cell receptor signaling, and Toll-like receptor signaling were reduced in the high-risk subgroup (Fig. 5A). GSEA results from various datasets suggested that the pathways of T-cell receptor signaling, natural killer cell-mediated cytotoxicity, and Toll-like receptor signaling were elevated in the low-risk score class (Fig. 5B and Supplementary Fig. 4). Moreover, the ESTIMATE algorithm determined that the high-risk group had lower immune scores, suggesting decreased immune cell infiltration in the tumor microenvironment (Fig. 5C and Supplementary Fig. 5). CIBERSORT analysis further revealed significant differences in the levels of infiltrating immune cells across the low-risk and high-risk score groups (Fig. 5D and Supplementary Fig. 6). The lowered abundance of immune-killing cells in the elevated-risk class implied the presence of an immunosuppressive tumor microenvironment, aligned with the documented negative prognosis.

### Screening for hub genes negative with immune

**Fig. 6:**
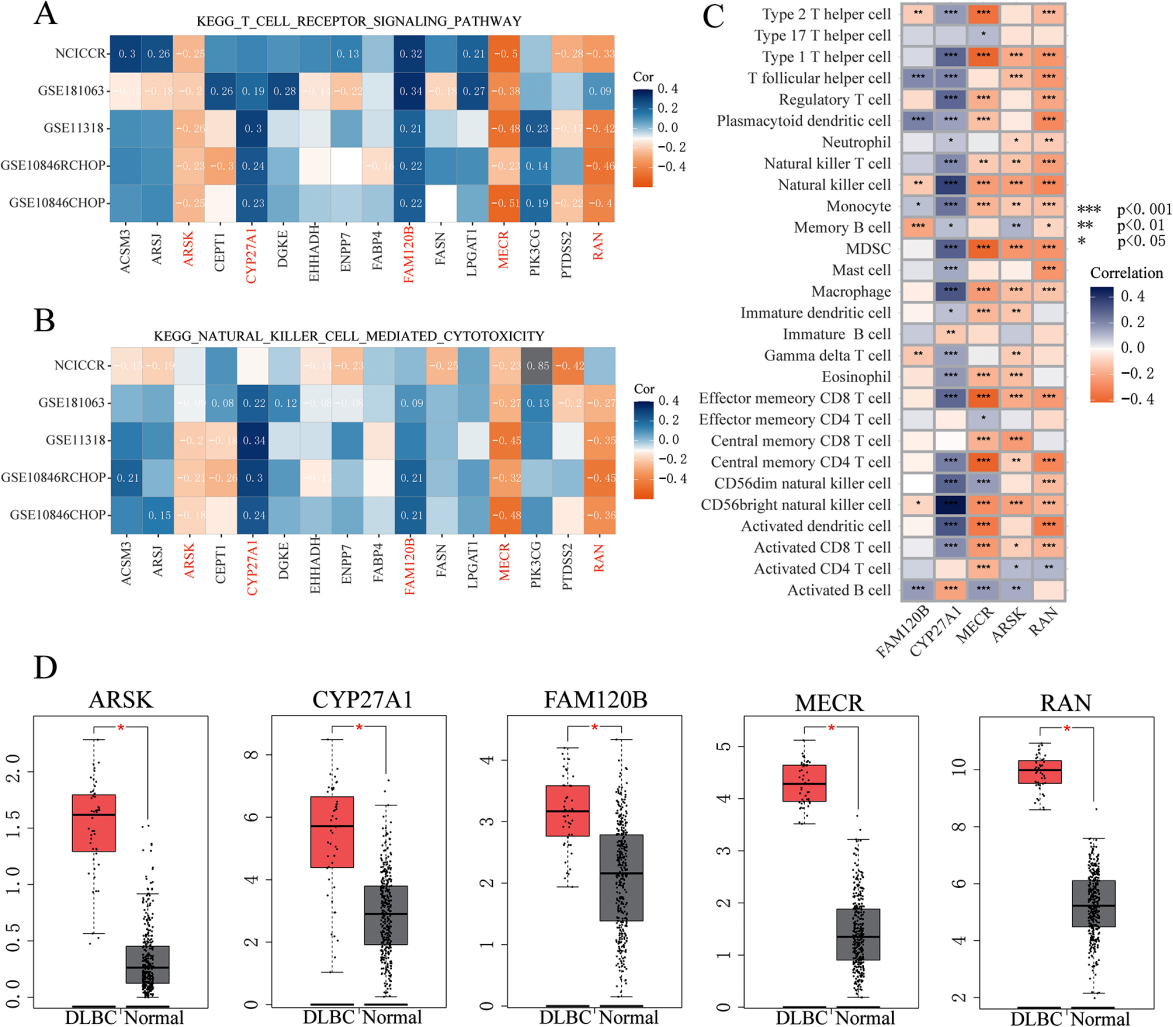
Identification of Immune-Independent Hub Genes. **(A)** Correlation analysis of the 16 genes associated with the T-cell receptor signaling pathway across multiple datasets, including GSE181063, GSE10846 R-CHOP, GSE10846 CHOP, GSE11318, and NCICCR. **(B)** Examination of the correlation between the 16 genes and natural killer cell-mediated cytotoxicity in the same datasets as in **(A)**. **(C)** Pearson correlation analysis illustrating the relationship between the expression of the 5 lipid metabolism/survival-related genes and the levels of infiltrating immune cells in the GSE181063 dataset. **(D)** Box plots displaying the expression patterns of the 5 genes analyzed using the GEPIA website. DLBCL patients are represented in red, while normal controls are depicted in grey. *P < 0.05

Given the relationship between lipid metabolism-derived risk level and the immune response, ssGSEA analysis was employed to uncover critical LMAGs influencing immune responses. *MECR*, *RAN*, and *ARSK* exhibited negative correlations with the T-cell receptor signaling pathway as well as natural killer cell-mediated cytotoxicity, while *CYP27A1* alongside *FAM120B* exhibited positive correlations with these pathways (Fig. 6A and B). Moreover, the immune infiltration examination of the GSE181063 dataset demonstrated that the levels of *MECR*, *RAN*, and *ARSK* were correlated with several immune cells (Fig. 6C). Comparable results were observed in GSE10846-CHOP, GSE10846-RCHOP, NCICCR, and GSE11318 datasets (Supplementary Fig. 7). Natural killer T cells, as the first line of defense against cancer, and CD8 T cells are considered as vital anti-tumor immune cells. Our results showed that the levels of *MECR*, *RAN*, and *ARSK* were negatively correlated with both activated CD8 T cells as well as natural killer T cells, indicating that these three genes could inhibit immune response. In addition, analysis utilizing the GEPIA database uncovered higher expression levels of *ARSK*, *CYP27A1*, *FAM120B*, *MECR*, and *RAN* in DLBCL patients in comparison to normal controls (Fig. 6D). By combining expression patterns with their effects on survival, it was postulated that *MECR*, *RAN*, and *ARSK* played significant parts in the poor development of DLBCL through modulation of the immune response.

### Clinical specimens’ verification

**Fig. 7:**
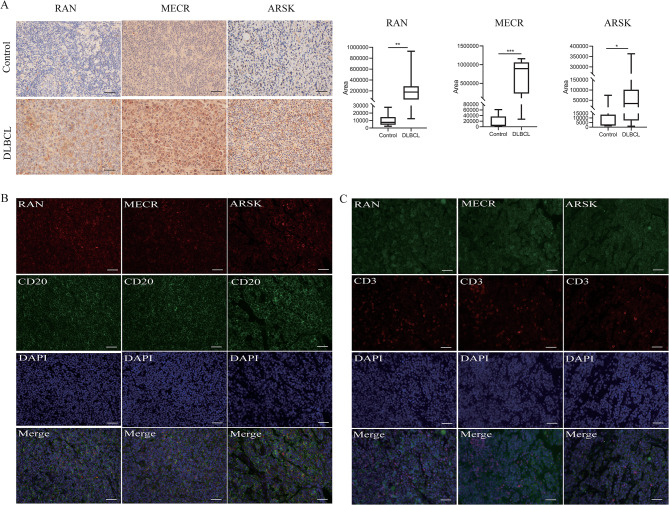
Clinical sample verification. **(A)** Microscopic images illustrating immunohistochemical staining for RAN, MECR, and ARSK in lymph node sections from both controls and DLBCL patients. Both images were captured at 400× magnification. The horizontal bar on the right demarcates the region displaying positive signal expression of RAN, MECR, and ARSK in the lymph node sections between these two groups. Scale bar corresponds to 200 pixels. ^***^P < 0.001; ^**^P < 0.01; ^*^P < 0.05. **(B-C)** Selected immunofluorescent photograph depicting the expression of RAN, MECR, and ARSK alongside the marker for lymphoma cell (CD20 in B) or the T cell (CD3 in C) in lymph node sections obtained from DLBCL patients. DAPI was utilized for nuclear staining (bar = 500 pixels)

To validate the expression of *MECR*, *RAN*, and *ARSK* in clinical specimens, lymph node biopsies from DLBCL and lymphoid hyperplasia patients were assessed. Immunohistochemistry demonstrated significantly elevated expression of *MECR*, *RAN*, and *ARSK* in DLBCL patients (Fig. 7A). Immunofluorescence analysis of DLBCL sections showed that every MECR + cell expressed CD20, and the same pattern was observed in RAN + cells or ARSK + cells, indicating the expression of these genes in lymphoma cells (Fig. 7B). Importantly, these three proteins were not observed to be expressed in CD3^+^ cells (Fig. 7C), suggesting that they may impact the immune response indirectly via tumor cells rather than directly manipulating T cells.

## Discussion

The molecular heterogeneity of DLBCL poses a significant obstacle to current precision therapy strategies. While the IPI scoring system, which uses clinical characteristics, has been valuable, it does not fully capture the complexity of DLBCL prognosis, especially for patients with low or high mortality risk. In this research, a robust lipid metabolism-associated risk score approach related to DLBCL patients has been revealed, which not only complements the limitations of the IPI score but also stands as an independent prognostic factor. Additionally, this analysis has uncovered three hub genes linked to an immunosuppressive microenvironment throughout the elevated-risk score individuals, shedding light on putative targets within lipid metabolic pathways for precise immunotherapy in DLBCL.

The reported risk score model consists of 16 lipid metabolism-related genes that have been previously investigated in solid cancers, with the exception of *ARSK*. Several of these genes have shown promise as potential prognostic biomarkers in cancer, such as *FAMA120B* in ovarian cancer [27], *ARSJ* in colorectal cancer, and *PTDSS2* in hepatocellular carcinoma [28, 29]. Many of these genes have been associated with cancer progression, proliferation, metastasis, inhibition of apoptosis, and chemotherapy resistance [29–38]. However, it is worth noting that *ACSM3* has demonstrated tumor-immunosuppressive properties in high-grade serous ovarian cancer, and *ENPP7* activity has been relatively low in diseases with an increased risk of liver tumorigenesis [39, 40]. Among the 16 genes, only *FASN* and *CYP27A1* have been previously reported in DLBCL [15, 16, 41, 42]. Nevertheless, the biological processes in which these 16 genes are involved have been well-established in DLBCL, including biosynthesis, transport, and beta-oxidation of fatty acids, as well as acyl-CoA flux within cells and lipid metabolism [16, 17, 43–45]. Therefore, the findings of this study support the potential of a predictive approach according to lipid metabolism for DLBCL.

The IPI score model, a widely used prognostic scoring system, classifies patients into four risk classes according to their complete score (from low, low-intermediate, high-intermediate, to high risk) [5]. This scoring system assigns one point to each negative prognostic factor, such as over 60 years old, increased LDH levels, stage-III/IV Ann Arbor disease, ≥ 2 ECOG performance status, and more than one site with extranodal involvement. The findings presented in this study demonstrate that the lipid metabolism-associated risk level may significantly discriminate the individual’s OS with DLBCL possessing low-risk or high-risk IPI scores. This suggests the need to integrate biological tumor features into existing clinical scoring systems. Additionally, this work provided evidence for low-risk IPI patients with high recurrence rates and high-risk IPI patients with long-term survival. The risk score model presented in this study has the potential to classify patients into six risk groups, enabling a new prognostic model in combination with the IPI.

In terms of the activities within the tumor immune microenvironment, this study exhibited the inhibition of several immune-related pathways throughout the elevated-risk score class, suggesting the presence of an immunosuppressive tumor microenvironment within DLBCL patients with elevated-risk scores. Furthermore, three hub genes linked to immunosuppression in DLBCL were identified. Those outside of *ARSK*, *MECR*, and *RAN* have been implicated as oncogenes in solid cancers [46–49]. The bioinformatics analysis demonstrated the high expression of these three genes within DLBCL. Moreover, the levels of these genes were negatively correlated with OS, suggesting potential roles as oncogenes in DLBCL. Immunofluorescence verified these gene expressions within lymphoma but not T cells, indicating that these genes may indirectly influence the immune response by modulating tumor cells. To date, there have been no reports on the direct impact of *MECR*, *ARSK*, and *RAN* on the immune response. The results presented suggest that these genes affect DLBCL prognosis by indirectly inhibiting the immune response. However, while it was documented that the function of circulatory NK cells was inhibited in DLBCL [50], the gene expression of NK cells was not assessed due to the limited number of NK cells in the lymph node paraffin sections of DLBCL. More studies are required to deepen the understanding how lipid metabolism-related genes affect immune cells.

### Strengths and limitations

This is the first report of an LMAGs-derived risk model for DLBCL patients, and it has been validated using multicenter public datasets. Moreover, the risk approach may further discriminate the diverse prognosis of DLBCL-patients into low-risk or high-risk IPI scores, complementing the limitations of the IPI score, and offering a new prognostic model for clinical judgment. Of course, limitations are present within this research. To begin with, the clinical information profiles from samples found in public databases are finite. The established nomogram should be validated using prospective clinical trials with a larger sample size. Second, the effect of any lipid metabolism-based risk score on progression-free survival (PFS) in DLBCL was not analyzed because of the limited availability of PFS data in public databases. Finally, further in vivo experiments will be considered to strengthen our results.

## Conclusions

A risk score model based on the lipid metabolism represents a robust prognostic signature for DLBCL, linked to an immunosuppressive tumor microenvironment. This risk score model has the ability to further stratify DLBCL patients, particularly those classified as low-risk or high-risk based on IPI groups. Overall, systematic investigation of lipid metabolism allows for insights into individual risk stratification and offers innovative perspectives for personalized therapy targeting DLBCL.

### Electronic supplementary material

Below is the link to the electronic supplementary material.

Supplementary Material 1

Supplementary Material 2

Supplementary Material 3

Supplementary Material 4

Supplementary Material 5

Supplementary Material 6

Supplementary Material 7

Supplementary Material 8

Supplementary Material 9

Supplementary Material 10

## Data Availability

All data examined during this study can be obtained from public databases.

## References

[1] Flowers CR, Sinha R, Vose JM (2010). Improving outcomes for patients with diffuse large B-cell lymphoma. CA Cancer J Clin.

[2] Reddy A, Zhang J, Davis NS, Moffitt AB, Love CL, Waldrop A, Leppa S, Pasanen A, Meriranta L, Karjalainen-Lindsberg ML (2017). Genetic and functional drivers of diffuse large B cell lymphoma. Cell.

[3] Chapuy B, Stewart C, Dunford AJ, Kim J, Kamburov A, Redd RA, Lawrence MS, Roemer MGM, Li AJ, Ziepert M (2018). Molecular subtypes of diffuse large B cell lymphoma are associated with distinct pathogenic mechanisms and outcomes. Nat Med.

[4] Schmitz R, Wright GW, Huang DW, Johnson CA, Phelan JD, Wang JQ, Roulland S, Kasbekar M, Young RM, Shaffer AL (2018). Genetics and pathogenesis of diffuse large B-cell lymphoma. N Engl J Med.

[5] International Non-Hodgkin’s Lymphoma Prognostic Factors Project (1993). A predictive model for aggressive non-hodgkin’s lymphoma. N Engl J Med.

[6] Stephens DM, Li H, LeBlanc ML, Puvvada SD, Persky D, Friedberg JW, Smith SM (2016). Continued risk of relapse independent of treatment modality in limited-stage diffuse large B-cell lymphoma: final and long-term analysis of southwest oncology group study S8736. J Clin Oncol.

[7] Ruppert AS, Dixon JG, Salles G, Wall A, Cunningham D, Poeschel V, Haioun C, Tilly H, Ghesquieres H, Ziepert M (2020). International prognostic indices in diffuse large B-cell lymphoma: a comparison of IPI, R-IPI, and NCCN-IPI. Blood.

[8] Cheng C, Geng F, Cheng X, Guo D (2018). Lipid metabolism reprogramming and its potential targets in cancer. Cancer Commun (Lond).

[9] Bian X, Liu R, Meng Y, Xing D, Xu D, Lu Z (2021). Lipid metabolism and cancer. J Exp Med.

[10] Broadfield LA, Pane AA, Talebi A, Swinnen JV, Fendt SM (2021). Lipid metabolism in cancer: new perspectives and emerging mechanisms. Dev Cell.

[11] Ma K, Zhang L (2021). Overview: lipid metabolism in the tumor microenvironment. Adv Exp Med Biol.

[12] Ye X, Zhang G, Righolt C, Johnston JB, Banerji V, Gibson SB, Mahmud SM (2018). Associations between statin use and risk of non-hodgkin lymphomas by subtype. Int J Cancer.

[13] Gouni S, Strati P, Toruner G, Aradhya A, Landgraf R, Bilbao D, Vega F, Agarwal NK (2022). Statins enhance the chemosensitivity of R-CHOP in diffuse large B-cell lymphoma. Leuk Lymphoma.

[14] Caro P, Kishan AU, Norberg E, Stanley IA, Chapuy B, Ficarro SB, Polak K, Tondera D, Gounarides J, Yin H (2012). Metabolic signatures uncover distinct targets in molecular subsets of diffuse large B cell lymphoma. Cancer Cell.

[15] Zhong X, Liu Z, Luo Q, Li J, Zhang W, Shuang Y (2021). Upregulation of fatty acid synthase in MYC and BCL-2 double-expressor lymphoma. Oncol Lett.

[16] Uddin S, Hussain AR, Ahmed M, Bu R, Ahmed SO, Ajarim D, Al-Dayel F, Bavi P, Al-Kuraya KS (2010). Inhibition of fatty acid synthase suppresses c-Met receptor kinase and induces apoptosis in diffuse large B-cell lymphoma. Mol Cancer Ther.

[17] Liu MK, Cheng LL, Yi HM, He Y, Li X, Fu D, Dai YT, Fang H, Cheng S, Xu PP (2022). Enhanced lipid metabolism confers the immunosuppressive tumor microenvironment in CD5-positive non-MYC/BCL2 double expressor lymphoma. Front Oncol.

[18] Zhu M, Zeng Q, Fan T, Lei Y, Wang F, Zheng S, Wang X, Zeng H, Tan F, Sun N (2022). Clinical significance and immunometabolism landscapes of a novel recurrence-associated lipid metabolism signature in early-stage lung adenocarcinoma: a comprehensive analysis. Front Immunol.

[19] Jiang C, Liu Y, Wen S, Xu C, Gu L (2021). In silico development and clinical validation of novel 8 gene signature based on lipid metabolism related genes in colon adenocarcinoma. Pharmacol Res.

[20] Bai R, Rebelo A, Kleeff J, Sunami Y (2021). Identification of prognostic lipid droplet-associated genes in pancreatic cancer patients via bioinformatics analysis. Lipids Health Dis.

[21] Zhu K, Xiaoqiang L, Deng W, Wang G, Fu B (2021). Development and validation of a novel lipid metabolism-related gene prognostic signature and candidate drugs for patients with bladder cancer. Lipids Health Dis.

[22] Ye Z, Zou S, Niu Z, Xu Z, Hu Y (2021). A novel risk model based on lipid metabolism-associated genes predicts prognosis and indicates immune microenvironment in breast cancer. Front Cell Dev Biol.

[23] Liberzon A, Subramanian A, Pinchback R, Thorvaldsdóttir H, Tamayo P, Mesirov JP (2011). Molecular signatures database (MSigDB) 3.0. Bioinformatics.

[24] Barrett T, Wilhite SE, Ledoux P, Evangelista C, Kim IF, Tomashevsky M, Marshall KA, Phillippy KH, Sherman PM, Holko M (2013). NCBI GEO: archive for functional genomics data sets–update. Nucleic Acids Res.

[25] Tomczak K, Czerwińska P, Wiznerowicz M (2015). The Cancer Genome Atlas (TCGA): an immeasurable source of knowledge. Contemp Oncol (Pozn).

[26] Chong Zhao R, Huang Z, Zeng S, Yang W, Lu J, Liu Y, Wei H, Guo Y, Zhang P (2021). Downregulation of USP18 reduces tumor-infiltrating activated dendritic cells in extranodal diffuse large B cell lymphoma patients. Aging.

[27] Cai J, Qiu J, Wang H, Sun J, Ji Y (2021). Identification of potential biomarkers in ovarian carcinoma and an evaluation of their prognostic value. Ann Transl Med.

[28] Mohammed M, Mboya IB, Mwambi H, Elbashir MK, Omolo B (2021). Predictors of colorectal cancer survival using cox regression and random survival forests models based on gene expression data. PLoS ONE.

[29] Liu J, Lu J, Li W (2021). A comprehensive prognostic and immunological analysis of a new three-gene signature in hepatocellular carcinoma. Stem Cells Int.

[30] Wang X, Luo G, Zhang K, Cao J, Huang C, Jiang T, Liu B, Su L, Qiu Z (2018). Hypoxic tumor-derived exosomal miR-301a mediates M2 macrophage polarization via PTEN/PI3Kγ to promote pancreatic cancer metastasis. Cancer Res.

[31] Li J, Kaneda MM, Ma J, Li M, Shepard RM, Patel K, Koga T, Sarver A, Furnari F, Xu B (2021). PI3Kγ inhibition suppresses microglia/TAM accumulation in glioblastoma microenvironment to promote exceptional temozolomide response. Proc Natl Acad Sci U S A.

[32] Nelson ER, Wardell SE, Jasper JS, Park S, Suchindran S, Howe MK, Carver NJ, Pillai RV, Sullivan PM, Sondhi V (2013). 27-Hydroxycholesterol links hypercholesterolemia and breast cancer pathophysiology. Science.

[33] Nieman KM, Kenny HA, Penicka CV, Ladanyi A, Buell-Gutbrod R, Zillhardt MR, Romero IL, Carey MS, Mills GB, Hotamisligil GS (2011). Adipocytes promote ovarian cancer metastasis and provide energy for rapid tumor growth. Nat Med.

[34] Okamura S, Yoshino H, Kuroshima K, Tsuruda M, Osako Y, Sakaguchi T, Yonemori M, Yamada Y, Tatarano S, Nakagawa M (2021). EHHADH contributes to cisplatin resistance through regulation by tumor-suppressive microRNAs in bladder cancer. BMC Cancer.

[35] Barlin JN, Jelinic P, Olvera N, Bogomolniy F, Bisogna M, Dao F, Barakat RR, Chi DS, Levine DA (2013). Validated gene targets associated with curatively treated advanced serous ovarian carcinoma. Gynecol Oncol.

[36] Ye Q, Raese R, Luo D, Cao S, Wan YW, Qian Y, Guo NL (2023). MicroRNA, mRNA, and proteomics biomarkers and therapeutic targets for improving lung cancer treatment outcomes. Cancers (Basel).

[37] Gong H, Ma C, Li X, Zhang X, Zhang L, Chen P, Wang W, Hu Y, Huang T, Wu N, Wang X (2023). Upregulation of LPGAT1 enhances lung adenocarcinoma proliferation. Front Biosci (Landmark Ed).

[38] Du Q, Liu P, Zhang C, Liu T, Wang W, Shang C, Wu J, Liao Y, Chen Y, Huang J (2022). FASN promotes lymph node metastasis in cervical cancer via cholesterol reprogramming and lymphangiogenesis. Cell Death Dis.

[39] Yang X, Wu G, Zhang Q, Chen X, Li J, Han Q, Yang L, Wang C, Huang M, Li Y (2022). ACSM3 suppresses the pathogenesis of high-grade serous ovarian carcinoma via promoting AMPK activity. Cell Oncol (Dordr).

[40] Banerjee S, Norman DD, Lee SC, Parrill AL, Pham TC, Baker DL, Tigyi GJ, Miller DD (2017). Highly potent non-carboxylic acid autotaxin inhibitors reduce melanoma metastasis and chemotherapeutic resistance of breast cancer stem cells. J Med Chem.

[41] Kapadia B, Nanaji NM, Bhalla K, Bhandary B, Lapidus R, Beheshti A, Evens AM, Gartenhaus RB (2018). Fatty acid synthase induced S6Kinase facilitates USP11-eIF4B complex formation for sustained oncogenic translation in DLBCL. Nat Commun.

[42] Pan M, Yang P, Wang F, Luo X, Li B, Ding Y, Lu H, Dong Y, Zhang W, Xiu B (2021). Whole transcriptome data analysis reveals prognostic signature genes for overall survival prediction in diffuse large B cell lymphoma. Front Genet.

[43] Sekine Y, Yamamoto K, Kurata M, Honda A, Onishi I, Kinowaki Y, Kawade G, Watabe S, Nomura S, Fukuda S (2022). HADHB, a fatty acid beta-oxidation enzyme, is a potential prognostic predictor in malignant lymphoma. Pathology.

[44] Li M, Chiang YL, Lyssiotis CA, Teater MR, Hong JY, Shen H, Wang L, Hu J, Jing H, Chen Z (2019). Non-oncogene addiction to SIRT3 plays a critical role in lymphomagenesis. Cancer Cell.

[45] Schmitt A, Xu W, Bucher P, Grimm M, Konantz M, Horn H, Zapukhlyak M, Berning P, Brändle M, Jarboui MA (2021). Dimethyl fumarate induces ferroptosis and impairs NF-κB/STAT3 signaling in DLBCL. Blood.

[46] Miinalainen IJ, Chen ZJ, Torkko JM, Pirilä PL, Sormunen RT, Bergmann U, Qin YM, Hiltunen JK (2003). Characterization of 2-enoyl thioester reductase from mammals. An ortholog of YBR026p/MRF1’p of the yeast mitochondrial fatty acid synthesis type II. J Biol Chem.

[47] Cai Y, Lin Y, Xiong X, Lu J, Zhou R, Jin Y, You Z, Ye H, Li F, Cheng N (2019). Knockdown expression of MECR, a novel gene of mitochondrial FAS II inhibits growth and colony-formation, promotes apoptosis of hepatocelluar carcinoma cells. Biosci Trends.

[48] Boudhraa Z, Carmona E, Provencher D, Mes-Masson AM (2020). Ran GTPase: a key player in tumor progression and metastasis. Front Cell Dev Biol.

[49] El-Tanani M, Nsairat H, Mishra V, Mishra Y, Aljabali AAA, Serrano-Aroca Á, Tambuwala MM (2023). Ran GTPase and its importance in cellular signaling and malignant phenotype. Int J Mol Sci.

[50] Kobayashi T, Lam PY, Jiang H, Bednarska K, Gloury R, Murigneux V, Tay J, Jacquelot N, Li R, Tuong ZK (2020). Increased lipid metabolism impairs NK cell function and mediates adaptation to the lymphoma environment. Blood.

