# Supplementary material for: A novel lipid metabolism-based risk model associated with immunosuppressive mechanisms in diffuse large B-cell lymphoma
Source: Lipids Health Dis. 2024 Jan 22;23:20. doi: 10.1186/s12944-024-02017-z (PMC10801940; doi:10.1186/s12944-024-02017-z)
Supplement: Supplementary file 1 — Supplementary Material 1 [file 12944_2024_2017_MOESM1_ESM.docx]

**Supplementary Table 1.** 776 genes implicated in lipid metabolism were extracted from the Molecular Signature Database (MSigDB).

**Supplementary Table 2.** 523 lipid metabolism-associated genes were assembled from the expression profiles originating from the Gene Expression Omnibus (GEO) database and The Cancer Genome Atlas (TCGA) database.

**Supplementary Figure Legends**

**Supplementary Figure 1.** Kaplan-Meier survival curve derived from 16 genes.

**Supplementary Figure 2.** (A) Time-dependent C-index plot of the risk score as well as 16 single LMAGs. (B) ROC analyses of survival derived from the risk score and 16 single LMAGs.

**Supplementary Figure 3. KM curves for high- and low-risk samples across different clinical characteristics.** KM curves from high- and low-risk samples derived from patients who were: older (age>60) (A); younger (age≤60) (B); without B symptoms (C); with B symptoms (D); in ABC types (E); in GCB types (F); in ECOG <2 (G); in ECOG≥2 (H); lacking extranodal involvement (I); possessing extranodal involvement (J); male (K); female (L); in IPI 0-2 (M); in IPI 3-5 (N); in LDH > UNL (O); in LDH ≤UNL (P); in stage III+IV(Q); in stage I+II (R).

**Supplementary Figure 4.** GSEA demonstrates significant enrichment in immune-related processes across the GSE10846 R-CHOP dataset (A), GSE10846 CHOP dataset (B), GSE11318 dataset (C), and NCICCR dataset (D).

**Supplementary Figure 5.** Immune score, stromal score, and estimate score throughout the high- and low-risk groups from the GSE10846 R-CHOP dataset (A), GSE10846 CHOP dataset (B), GSE11318 dataset (C), and NCICCR dataset (D).

**Supplementary Figure 6.** ssGSEA algorithm for the comparison of the score of different infiltrating immune cells in patients with DLBCL possessing high- and low-risk scores from the GSE10846 R-CHOP dataset (A), GSE10846 CHOP dataset (B), GSE11318 dataset (C), and NCICCR dataset (D). ****P* < 0.001; ***P* < 0.01; **P* < 0.05.

**Supplementary Figure 7.** Pearson correlation among 5 lipid metabolism- and survival-related genes as well as the infiltrating immune cells from the GSE10846 R-CHOP dataset (A), GSE10846 CHOP dataset (B), GSE11318 dataset (C), and NCICCR dataset (D).
