# Supplementary figures and images for: A novel lipid metabolism-based risk model associated with immunosuppressive mechanisms in diffuse large B-cell lymphoma
Source: Lipids Health Dis. 2024 Jan 22;23:20. doi: 10.1186/s12944-024-02017-z (PMC10801940; doi:10.1186/s12944-024-02017-z)

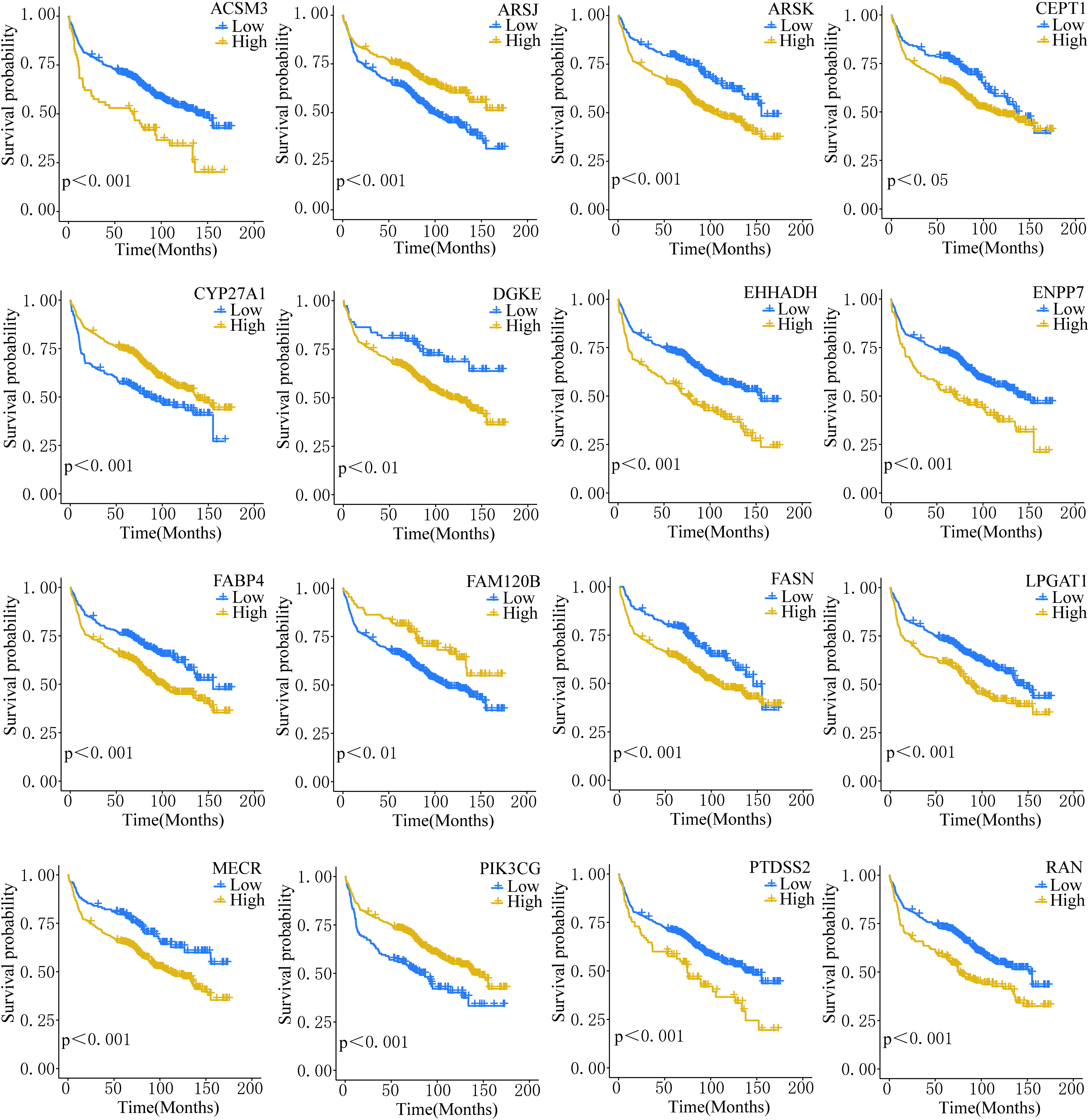

Supplement: Supplementary file 4 — Supplementary Material 4 [file 12944_2024_2017_MOESM4_ESM.tif]

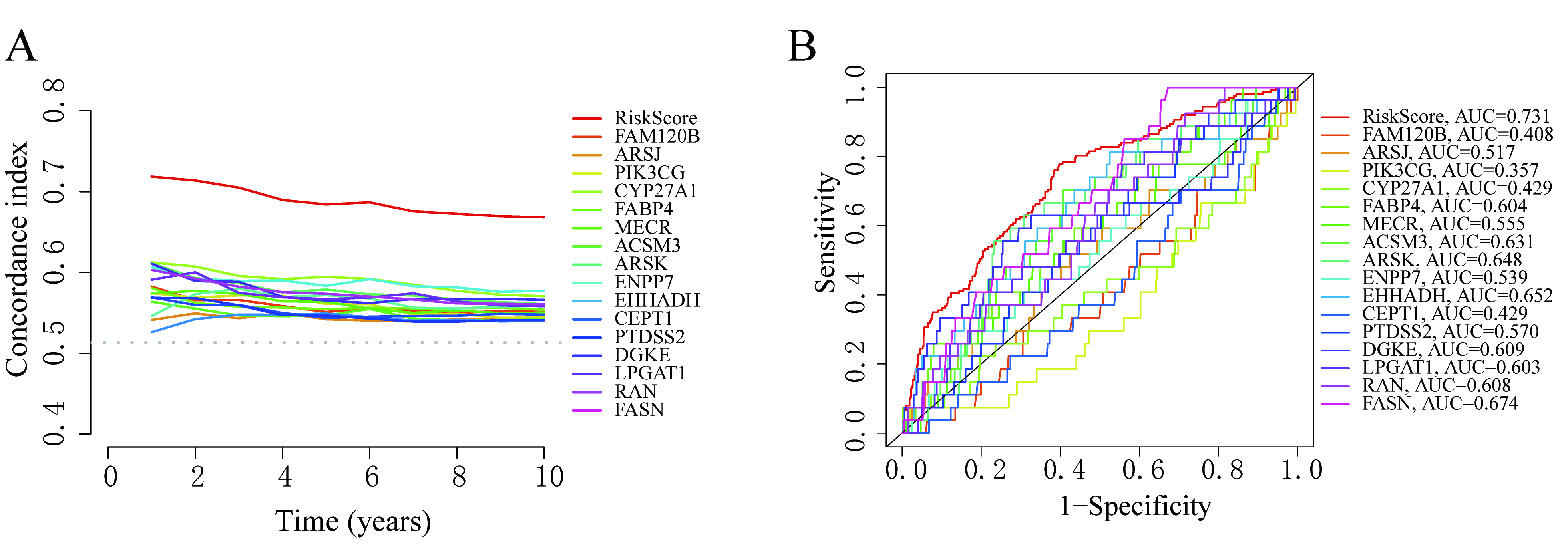

Supplement: Supplementary file 5 — Supplementary Material 5 [file 12944_2024_2017_MOESM5_ESM.tif]

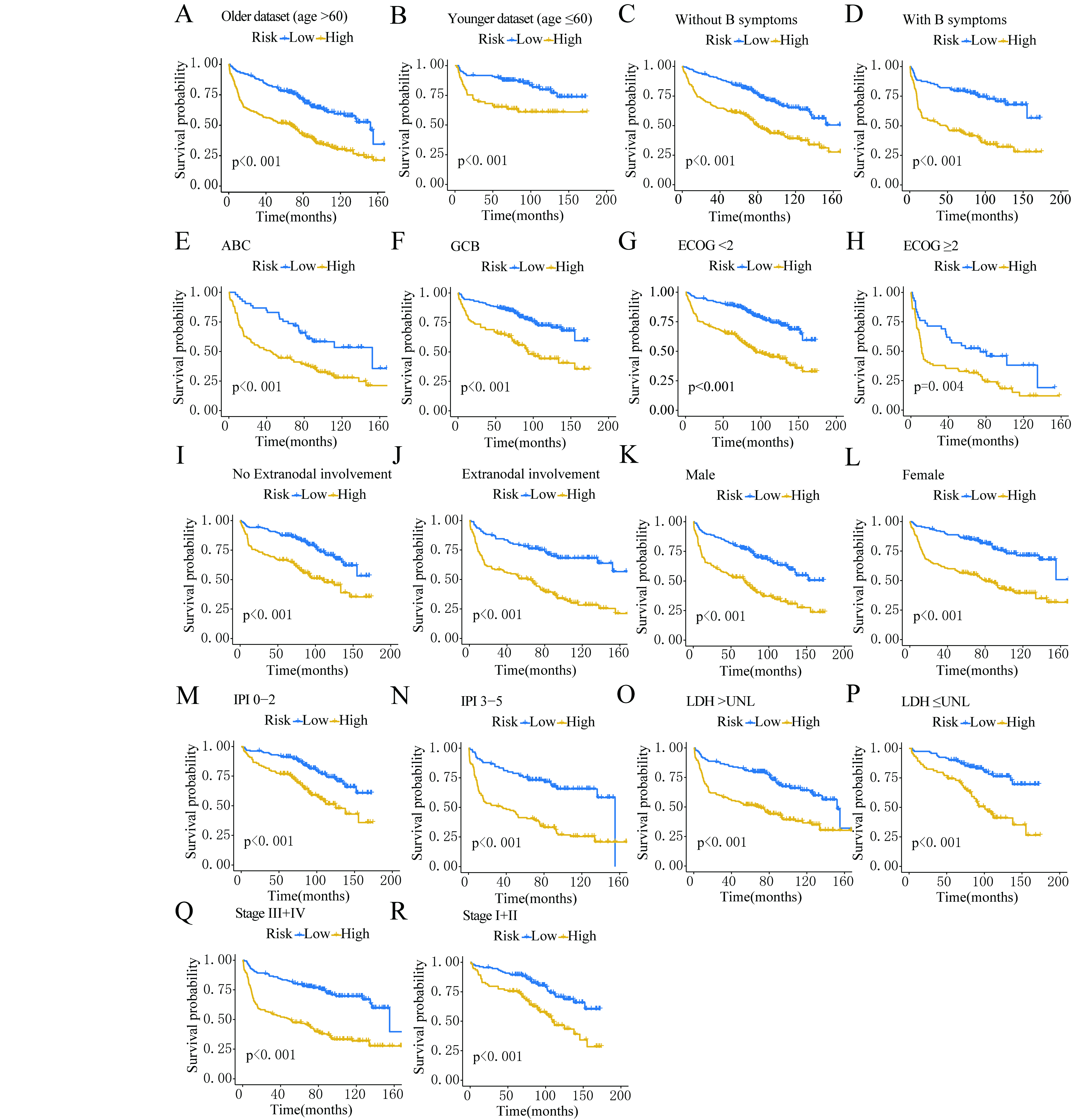

Supplement: Supplementary file 6 — Supplementary Material 6 [file 12944_2024_2017_MOESM6_ESM.tif]

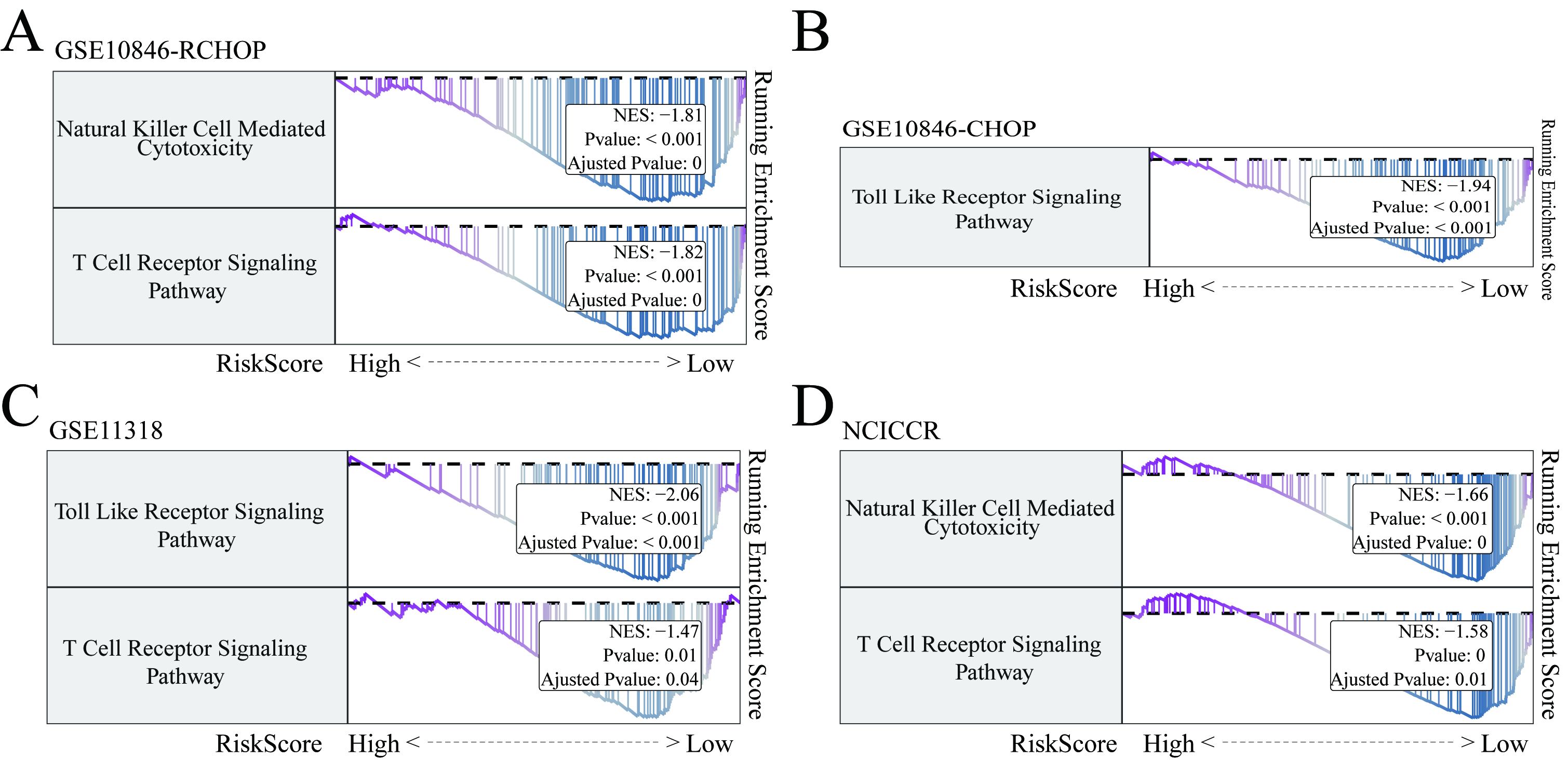

Supplement: Supplementary file 7 — Supplementary Material 7 [file 12944_2024_2017_MOESM7_ESM.tif]

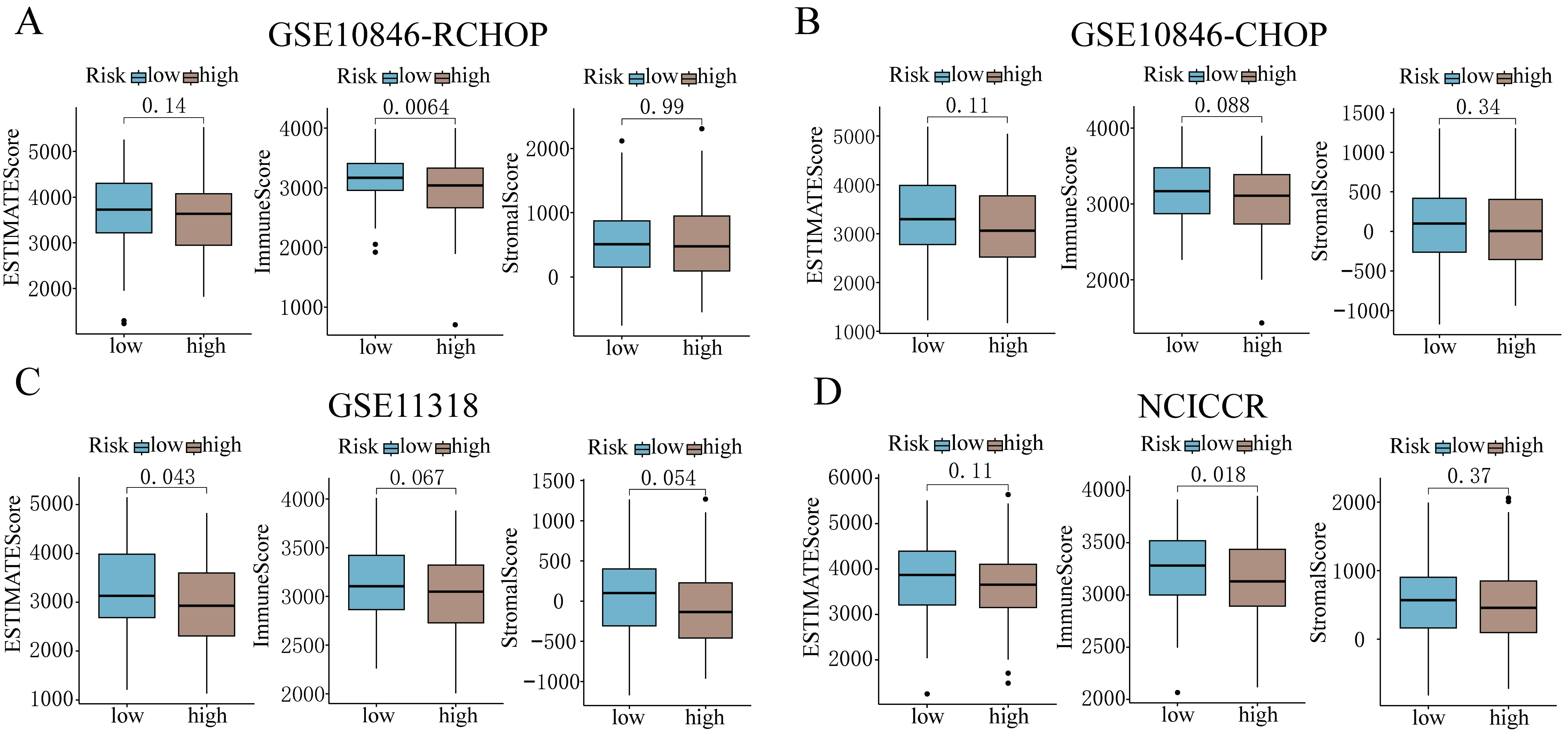

Supplement: Supplementary file 8 — Supplementary Material 8 [file 12944_2024_2017_MOESM8_ESM.tif]

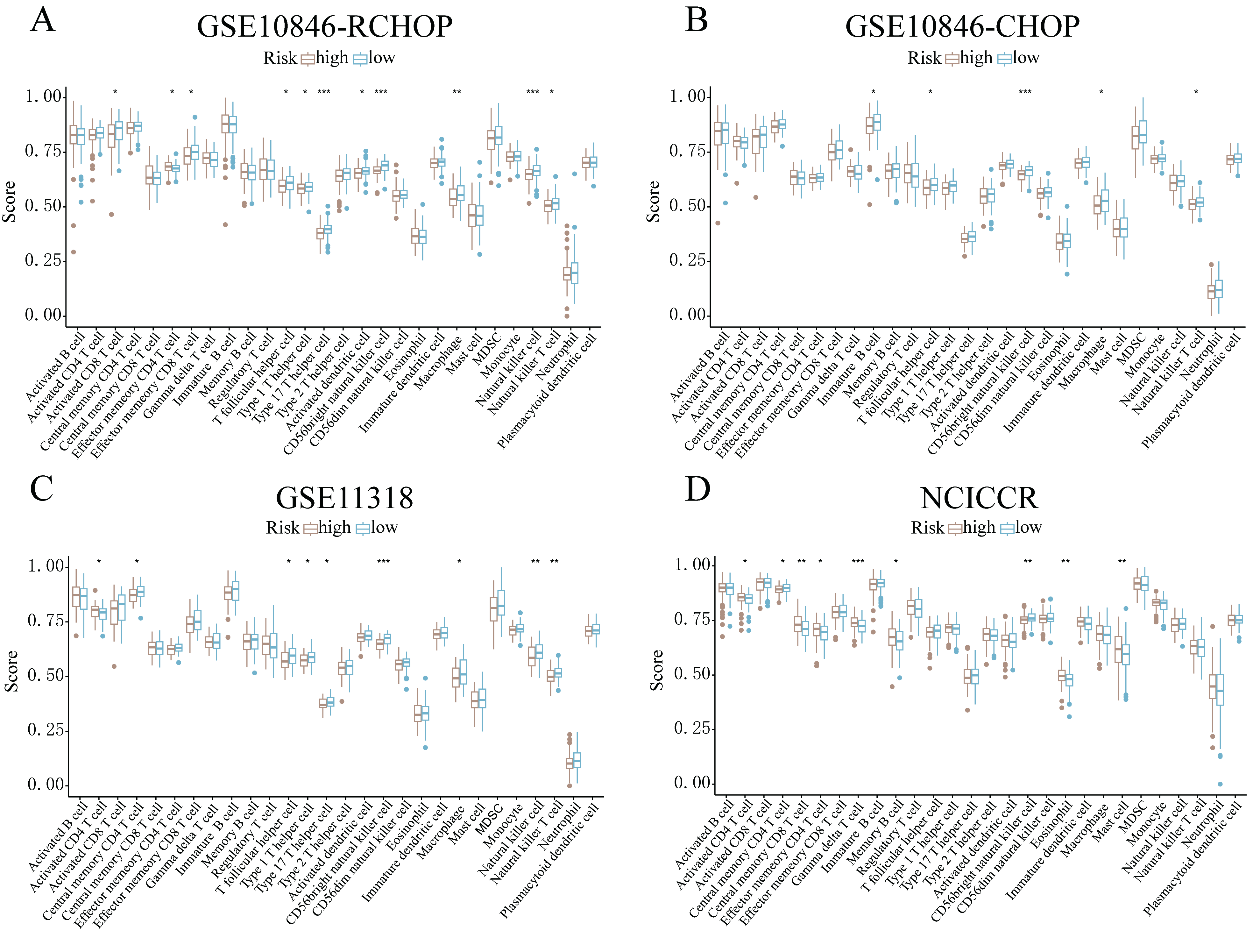

Supplement: Supplementary file 9 — Supplementary Material 9 [file 12944_2024_2017_MOESM9_ESM.tif]

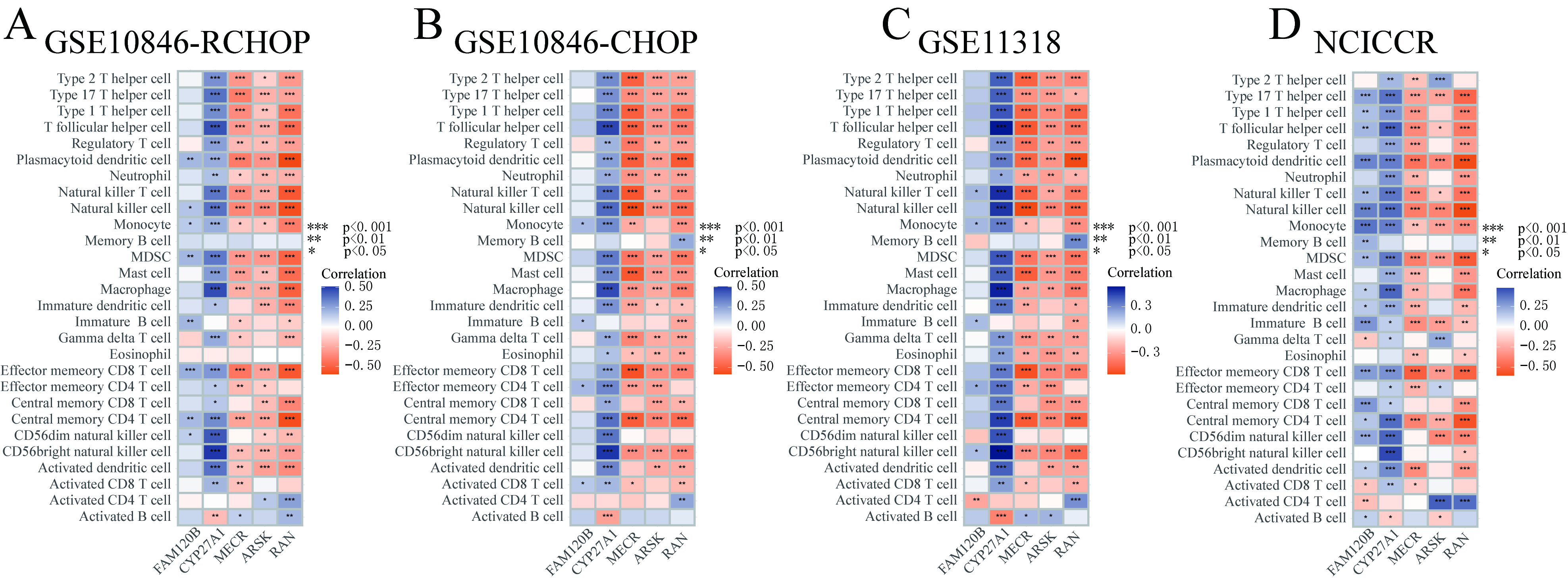

Supplement: Supplementary file 10 — Supplementary Material 10 [file 12944_2024_2017_MOESM10_ESM.tif]
